# Effects of Si Content and Ca Addition on Thermal Conductivity of As-Cast Mg–Si Alloys

**DOI:** 10.3390/ma11122376

**Published:** 2018-11-26

**Authors:** Xiong Zhou, Tian Guo, Shusen Wu, Shulin Lü, Xiong Yang, Wei Guo

**Affiliations:** State Key Laboratory of Materials Processing and Die & Mould Technology, Huazhong University of Science and Technology, 1037 Luoyu Road, Wuhan 430074, China; xiong_z@hust.edu.cn (X.Z.); guotian@hust.edu.cn (T.G.); shulin317@hust.edu.cn (S.L.); d201577265@hust.edu.cn (X.Y.); weiguo@hust.edu.cn (W.G.)

**Keywords:** Mg-Si alloy, modification, thermal conductivity, microstructure

## Abstract

The Mg–Si alloys have low CTEs (coefficients of thermal expansion) and other merits, which contribute to their application potential in the electronic industry. However, the details of their thermal properties are still unclarified, and need more research. In this study, the thermal conductivities (TC) of Mg–*x*Si (*x* = 1 wt.%, 1.38 wt.%, 2 wt.%, 3 wt.%, and 4 wt.%) binary alloys and Mg–4Si–*y*Ca (*y* = 0.2 wt.%, 0.4 wt.%, 0.6 wt.%, 0.8 wt.%, and 1.0 wt.%) alloys over the temperature range of 25–300 °C were systematically studied. The results show that the TC of Mg–*x*Si binary alloys decreases with the increase of Si content, while it increases slightly near the eutectic composition. The addition of a Ca element to Mg–4Si alloys has an obvious modification effect on the Mg_2_Si phase. When the Ca content increases constantly, the TC of the alloys decreases at first; then, there is a significant increase at the content of 0.8 wt.% Ca, and after that, it continues to decline. The mechanism is mainly related to the precipitation of the CaMgSi phase at 0.8 wt.% Ca content.

## 1. Introduction

As a new type of green structural material, magnesium alloys have wide applications because of their superior properties. There are a lot of research studies about the mechanical properties of magnesium alloys at present [1,2,3]. With the development of electronic products and the aerospace industry, the thermal properties of light metals, especially thermal conductivity (TC), are also required. The TC of pure magnesium is 157 W/(m·K) at room temperature, which is lower than copper and aluminum, but the magnesium alloy has the advantage of light weight; thus, the TC is larger per unit mass [4]. However, the magnesium alloy has a significant decrease in TC after adding common alloying elements. As a representative of commercial magnesium alloys, the TC of the AZ91D Mg alloy is only 51.2 W/(m·K). Therefore, new magnesium alloys need to be developed in order to meet thermal property requirements.

The TC characterizes the ability of the alloy to conduct heat. There are a large number of free electrons in the metal. The rapid movement of free electrons can achieve rapid heat transfer. Therefore, metals have good TC generally. In addition, the lattice waves can also act as a carrier of thermal energy. It is commonly assumed that the transport of heat in the alloy consists of two parts: electronic transmission and lattice transmission [5].

At present, there are many research studies on the TC of binary magnesium alloys. Ying [6,7] measured the TC of Mg–Zn binary alloys and Mg–Al binary alloys. It was confirmed that the TC of as-cast Mg–Zn alloys at room temperature decreased from 142 W/(m·K) to 110 W/(m·K), when the addition of Zn increased from 0.5 wt.% to 5.0 wt.%. The TC of Mg–Al binary alloys also fell down with an increase of Al content. For every per 0.5 wt.% increase of Al, the TC of alloys at room temperature decreases by nearly 15 W/(m·K). As the Zn or Al content increases, the volume of the precipitated second phase and the amount of the solid solution atom increase. This enhances the scattering of electrons and phonons, leading to a decrease in the TC of these alloys. Zhong [8] studied the change of TC of Mg–RE (rare earth) binary alloys by adding RE elements such as Ce, Nd, Y, or Gd. It was pointed out that the order of influence of four RE elements on the thermal resistance of the Mg alloy was Ce < Nd < Y < Gd. Due to its low solid solubility in the α–Mg phase, Ce has the weakest influence on the TC of the binary alloys.

Peng [9] added various concentrations of Ce to a ZM21 alloy and studied its influence on TC. It was shown that the TC of the alloy had an increase when 0.2 wt.% Ce was added, and then decreased gradually when the Ce element was further added. This is ascribed to the formation of MgZnCe and Mg_17_Ce_2_ phase in the alloy caused by the addition of Ce, which reduces the Zn and Ce content in the α–Mg phase. As a result, the lattice distortion is released, and the resistance to the movement of electrons and phonons is reduced, which leads to an improvement in the TC of the alloy. However, there is an increase in the amount of solid solution atoms with higher Ce content.

Magnesium has a high CTE (coefficient of thermal expansion), while a lower CTE is required in application fields such as the electronics industry. As stated above, studies on the TC of Mg alloys focus mostly on Mg–Zn [10,11], Mg–Al [12,13], and Mg–Sn [14,15] alloy systems, while there is almost no study on the thermal properties of Mg–Si alloys in which the precipitation of Mg_2_Si can effectively lower the CTE compared with pure magnesium. Furthermore, Mg–Si alloys have low cost, a good high-temperature stability, and excellent damping performance. The Si element has a small solid solubility in magnesium. Therefore, research on the TC of the Mg–Si binary alloys is meaningful and necessary.

Since the Mg_2_Si phase in Mg–Si alloys has a coarse morphology and degrades the mechanical properties of the alloy, it needs to be modified in practical applications. There are many studies on the modification of Mg_2_Si phase by adding alloying elements [16,17,18]. However, most of them focus on mechanical properties, and research on the thermal properties of the Mg–Si alloy is very scarce. Therefore, in the present study, it is necessary to investigate the effects of the addition of modification elements on the TC behavior, such as the addition of Ca.

## 2. Experimental Procedures

The experimental design of the alloys was Mg–*x*Si binary alloys (*x* = 1 wt.%, 1.38 wt.%, 2 wt.%, 3 wt.%, and 4 wt.%), and Mg–4Si–*y*Ca alloys (y = 0.2 wt.%, 0.4 wt.%, 0.6 wt.%, 0.8 wt.%, and 1.0 wt.%). The alloys were prepared by melting raw materials of pure 99.9 wt.% Mg, Mg–5 wt.% Si, and Mg–30 wt.% Ca master alloys in a resistance furnace. The whole melting process was under the protective atmosphere of mixed SF_6_ and N_2_, in which the mixture ratio of SF_6_ is 0.1 vol.%. The alloys were melted at 780 °C, and then the melts were degassed with high-purity Ar gas for five minutes. After removing the slag, the melt was held at 730 °C for 30 minutes, and then poured into a metallic mold that was preheated to 200 °C.

The specimens for microstructural observation were taken from the middle position of the ingots, and then polished and etched by 4 vol.% nital. The microstructure was observed under an optical micrograph (OM, CAIKON DMM-490C, caikon, Shanghai, China) and a scanning electron micrograph (SEM, JEOL-7600F, JEOL, Tokyo, Japan) equipped with an X-Max 50 type Energy Dispersive Spectroscopy(EDS). The software Image-Pro Plus was taken to analyze the phase volume fraction. The identification of the phases of the alloy was conducted with X-ray diffraction (XRD, Philips X’ pert, PANalytical, Almelo, Holland) with a copper target at a scanning speed of 10°/min from 10° to 90°.

The samples for the measurement of the thermal properties were a round disk with a thickness of 2.5 mm and a diameter of 12.5 mm. The thermal diffusion coefficient *k* of the alloys was measured by a Netzsch LFA427 flash analyzer (Netzsch, Selb, Germany). The room temperature densities of samples were identified by the Archimedes method, while their densities at high temperature were determined by following Equation (1) [19]:

(1) $$\rho =\rho_{0}-0.156\times \left(T-298\right)$$

where $\rho_{0}$ is the room temperature density, and *T* is the thermodynamic temperature.

The specific heat capacities of pure Mg, Si, and Ca element over the temperature range 25–300 °C can be expressed by Equations (2)–(4), respectively [20]:

(2) $$C_{p}\left(Mg\right)=0.89+4.58\times 10^{-4}T$$

(3) $$C_{P}\left(Si\right)=0.69+4.58\times 10^{-4}T$$

(4) $$C_{p}\left(Ca\right)=0.55+3.01\times 10^{-4}T$$

Therefore, the specific heat capacity of the alloys could be calculated by the Neumann–Kopp rule [21] in this study. The specific heat capacity and the density of alloys at room temperature are shown in Table 1 and Table 2.

The TC of alloys could be calculated with the following Equation (5):

(5) $$\lambda =k\rho C_{p}$$

where *λ* is the thermal conductivity, *k* is the thermal diffusivity, *ρ* is the density, and *C*_p_ is the specific heat capacity at constant pressure.

## 3. Results and Discussion

### 3.1. Microstructure and Thermal Conductivity of Mg–Si Binary Alloys

**Figure 1:**
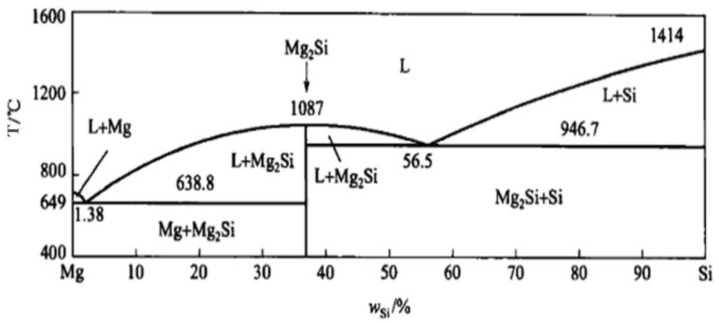
Mg–Si binary phase diagram.

**Figure 2:**
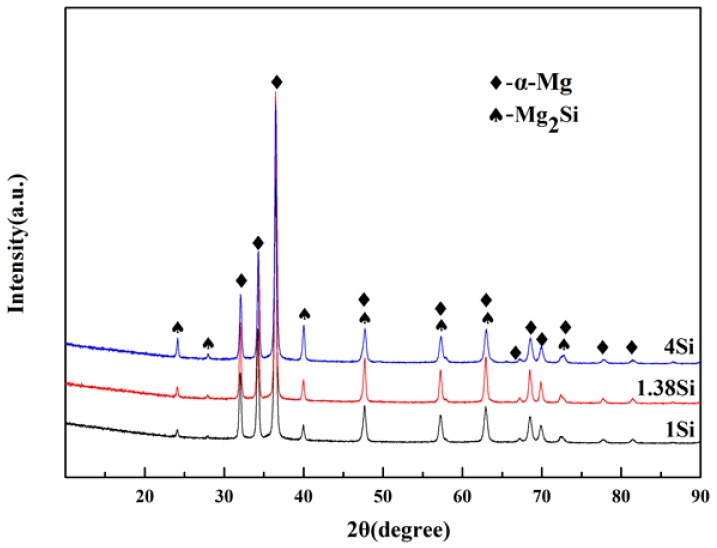
The XRD pattern for Mg–Si binary alloys.

**Figure 3:**
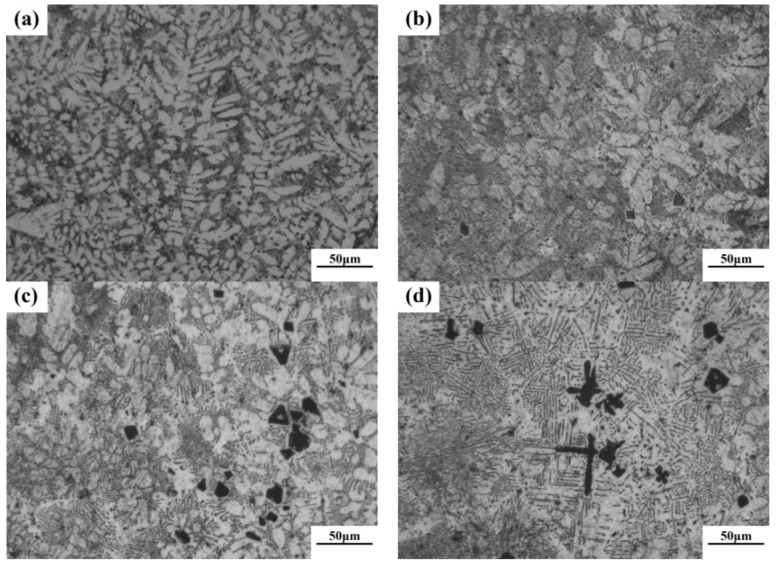

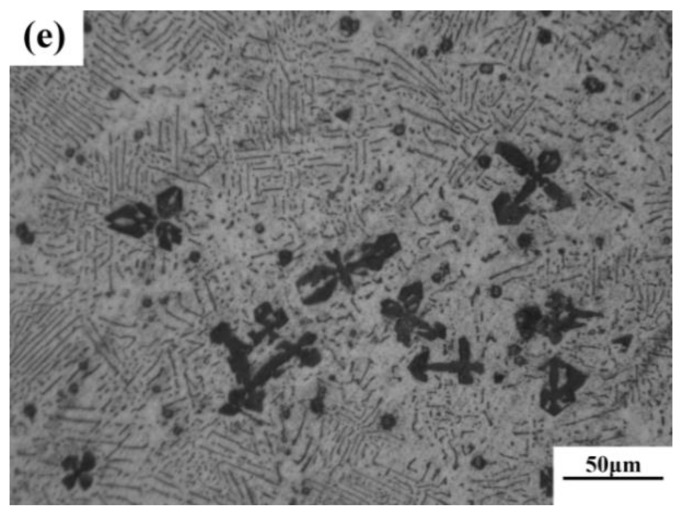
Optical microstructure of as-cast Mg–Si binary alloy. (**a**) Mg–1 wt.% Si; (**b**) Mg–1.38 wt.% Si; (**c**) Mg–2 wt.% Si (**d**) Mg–3 wt.% Si; (**e**) Mg–4 wt.% Si.

According to phase diagram shown in Figure 1, the Mg_2_Si phase is formed in the Mg–Si alloy during solidification. In this study, the phases of Mg–xSi alloys detected by XRD are shown in Figure 2. The Mg–Si binary alloys consisted of mainly α–Mg and Mg_2_Si phases. Figure 3 presents the optical microstructures of as-cast Mg–Si alloys with different Si content. It contains an α–Mg matrix and grey eutectic Mg_2_Si phase in the hypoeutectic Mg-1%Si alloy (Figure 3a). In addition to grey eutectic Mg_2_Si phase, small amount of black primary Mg_2_Si phase appeared in the eutectic composition of Mg–1.38%Si (Figure 3b). When the Si content was further increased, the primary Mg_2_Si phase coarsened gradually (Figure 3c–e).

**Figure 4:**
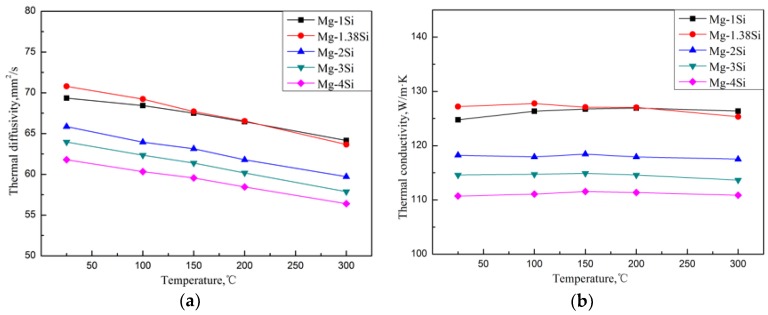
Thermal diffusivity (**a**) and thermal conductivity (**b**) of Mg–Si binary alloys.

Figure 4 shows the thermal diffusivities of the Mg–Si alloys, which were obtained by the flash method from 25 °C to 300 °C. According to Equation (5), the corresponding TC of the alloys from 25 °C to 300 °C can be obtained. Compared with pure magnesium, whose TC is 157 W/(m·K) at room temperature, the addition of Si causes a significant decrease in the TC. When Si content was increased from 1.0 wt.% to 4.0 wt.%, the TC of as-cast Mg-Si alloys decreased from 124.7 W/(m·K) to 110.7 W/(m·K) at room temperature. The reason is that, on the one hand, it is attributed to the lattice distortion of the Mg matrix, which is caused by the solid solution of Si atoms, and reduces the heat transportation of electrons and phonons. On the other hand, the Mg_2_Si phase introduces a large number of phase interfaces and enhances the scattering of electrons and phonons.

Referring to Ying’s research [6,7], the TCs of the Mg-1%Zn alloy and Mg-0.9%Al alloy were 138 W/(m·K) and 114 W/(m·K) at room temperature respectively. The decrement in TC with the addition of Si was greater than that with Zn and smaller than that with Al. The eutectic Mg_2_Si phase in the Mg–Si alloy is in the shape of a Chinese script character, while the second phase in the Mg–Zn alloy is in the form of particles or short rods. Thus, the Mg_2_Si phase in the Mg–Si alloy introduces more interfaces in the microstructure. Furthermore, Si atoms cause a greater distortion of the Mg lattice than Zn due to the difference in valence. Therefore, the addition of Si causes a greater decrement in TC. The solid solubility of Si atoms in the α–Mg phase is lower than that of Al atoms. Therefore, the lattice distortion of the α–Mg matrix generated by the addition of Si is not so severe compared with the addition of Al, which has less influence on the TC than Al.

It appears that the TC of the Mg–Si alloys has a tiny increase in the eutectic composition (1.38 wt.% Si) at room temperature, while the further addition of Si decreases the TC gradually (Figure 4). In order to discuss the change in TC in more detail, the volume fraction of the second phase in the Mg–Si binary alloys was measured, as shown in Table 3. It is assumed that the amount of the Mg_2_Si phase increases with the increase in Si content, which can be also observed in the XRD pattern shown in Figure 2. However, the volume fraction of the Mg_2_Si phase in the Mg-1.38%Si alloy is almost the same with that of the Mg-1%Si alloy, because of the similar composition.

The TC of metals has a close relationship with their electric conductivities. In metals, the vibrational contribution to the thermal conductivity is negligible compared to the electronic one (about 10%); then, according to Wiedemann–Franz law [4]:

(6) $$\lambda \rho =2.44\times 10^{-8}T$$

In which *λ* is the thermal conductivity, and *ρ* is the electrical resistivity. In the case of normal metals, the different contributions to the electrical resistivity are additive, and *ρ* can be thus expressed as Matthiessen’s rule [22]:

(7) $$\rho =\rho_{T}+\rho_{i}+\rho_{d}$$

where *ρ* is the total electrical resistivity; and *ρ_T_*, *ρ_i_* and *ρ_d_* are that of temperature, impurity, and deformation contributions, respectively. *ρ_T_* is a constant at a certain temperature, and *ρ_d_* can be ignored in the as-cast alloys. Impurity resistivity is mainly derived from solid-solution atoms and precipitated phases. For solid solution, electrical resistivity *ρ_i_*_1_ can be represented as follows:

(8) $$\rho_{i1}=Ac_{i}\left(1-c_{i}\right)$$

In which *A* is a constant related to impurity and the metal matrix, and $c_{i}$ is an impurity concentration (atom fraction). When $c_{i}$ < 50%, electrical resistivity increases with increasing impurity concentration. The impurity resistivity caused by precipitated phases is positively correlated with the volume fraction of the precipitated phases.

**Figure 5:**
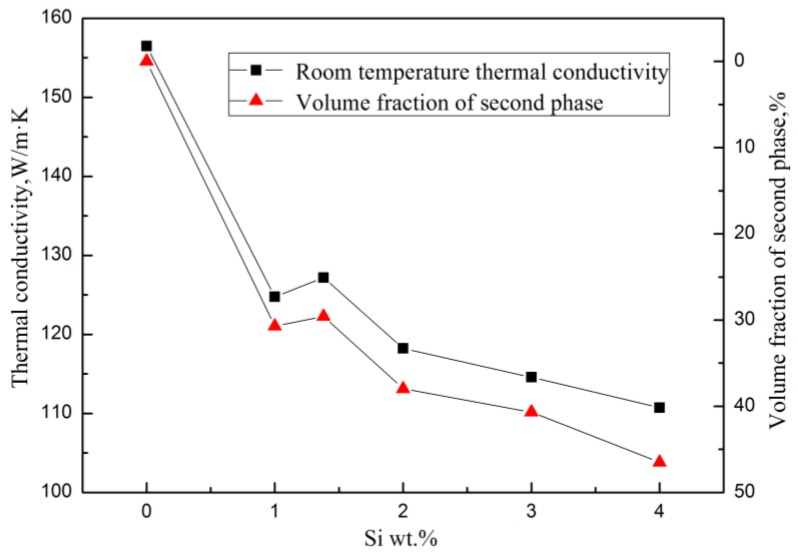
Relationships between room temperature thermal conductivity and volume fraction of the second phase of Mg–xSi alloys with Si content.

The Mg_2_Si has a very low TC, which is only 7.8 W/(m·K) at room temperature [23]. In the Mg–Si binary alloy, the α–Mg matrix is the main contributor to heat conduction. When the Si content was continuously increased, the amount of solid solution atoms and the volume fraction of the Mg_2_Si phase were also continuously increased, which enhanced the electrical resistivity of the alloy, making the heat transfer process more difficult. As a result, the TC of the alloy decreases significantly with the increase of Si content. It can be seen in Figure 5 that the thermal conductivity of the alloy has the same trend as the volume fraction of the second phase with various Si content at a certain temperature.

In the eutectic composition alloy (Mg–1.38%Si), because of non-equilibrium solidification, a certain amount of primary Mg_2_Si phase was generated, which consumed the Si in the alloys. Thus, the volume fraction of the eutectic Mg_2_Si phase is relatively reduced compared to the Mg–1%Si alloy. The electrical resistivity of the alloy can be affected by two factors—the amount of solid solution atoms and the volume fraction of precipitated phases—in which solid solution plays a more important role. It leads to a certain contribution to the enhancement in the TC of the Mg–1.38Si alloy compared with the Mg-1Si alloy, as indicated in Figure 4.

### 3.2. Modification Effect of Ca on Mg-4Si Alloy and Its Effect on Thermal Conductivity

**Figure 6:**
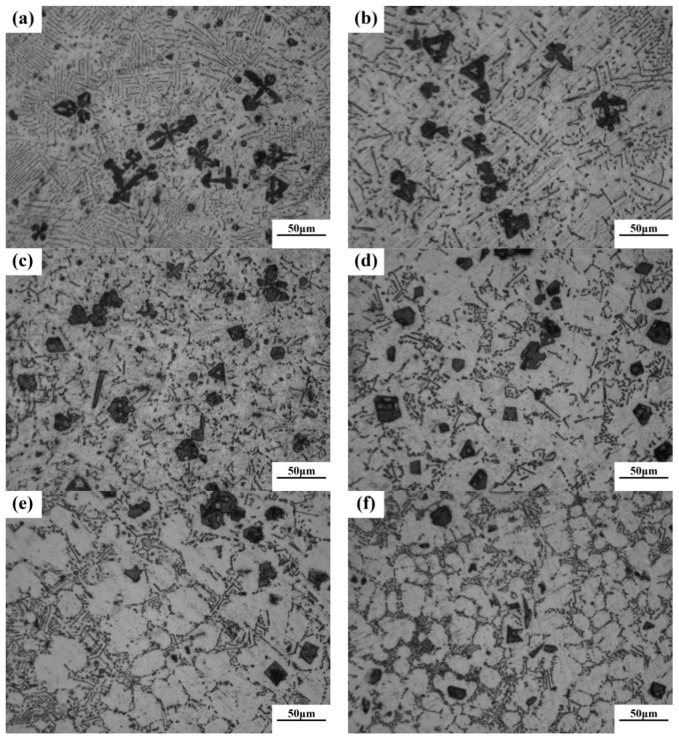
The optical micrograph (OM) images of as-cast Mg–4Si–yCa (y = 0 wt.%, 0.2 wt.%, 0.4 wt.%, 0.6 wt.%, 0.8 wt.%, 1.0 wt.%). (**a**) 0% Ca; (**b**) 0.2% Ca; (**c**) 0.4% Ca; (**d**) 0.6% Ca; (**e**) 0.8% Ca; (**f**) 1.0% Ca.

**Figure 7:**
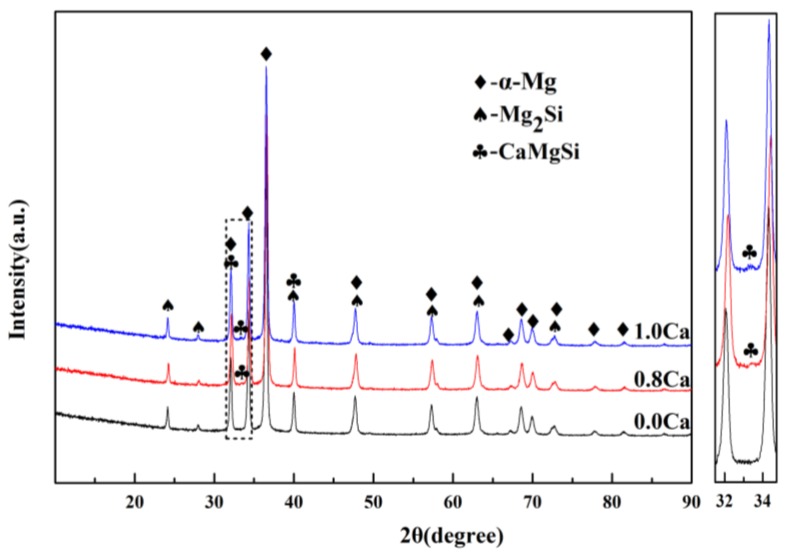
The XRD pattern of Mg–4Si–yCa (y = 0 wt.%, 0.8 wt.%, 1.0 wt.%).

**Figure 8:**
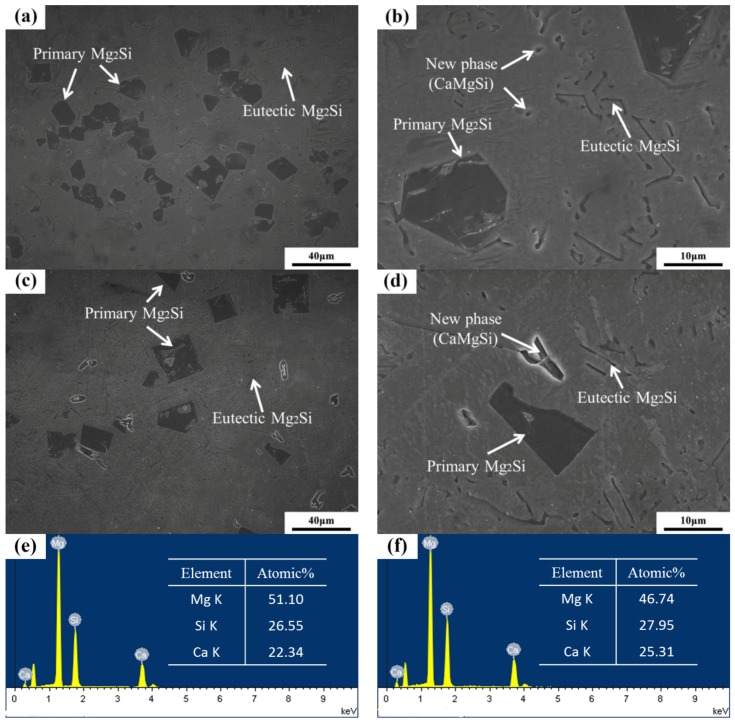
SEM images of Mg–4Si–0.8Ca and Mg–4Si–1.0Ca: (**a**,**b**) 0.8 wt.% Ca; (**c**,**d**) 1.0 wt.% Ca; (**e**) EDS of the new phase in the 0.8 wt.% Ca; (**f**) EDS of the new phase in the 1.0 wt.% Ca.

Figure 6 reveals the OM images of as-cast Mg–4Si–yCa alloys with various Ca contents. It is indicated that the phase composition of the Mg–4Si alloy is an α–Mg matrix, Chinese script eutectic Mg_2_Si phase, and coarse dendritic primary Mg_2_Si phase (Figure 6a). As shown in Figure 7, the phase constitutions are also confirmed by the XRD results. With the increase of Ca contents, the morphology of the primary Mg_2_Si phase changes from coarse dendritic (Figure 6a) to polygonal particles (Figure 6b–f). The size also decreases obviously, and the distribution of the eutectic Mg_2_Si phase becomes discontinuous. In addition, there are some new phases in the microstructure when the Ca content equals or exceeds 0.8 wt.%. Figure 8a,b are SEM images of alloys when adding 0.8 wt.% Ca. Some white fine particles appear in the alloy. When the Ca content reaches 1.0 wt.% (Figure 8c,d), new precipitated phases grow to a white needle-like shape. The corresponding EDS analysis of the new phase (Figure 8e,f) found that it is mainly composed of three elements: Mg, Si, and Ca. The Ca–Si ratio is close to 1:1. Moreover, the characteristic peak of the CaMgSi phase can be found in the XRD pattern (Figure 7). The new phase has a low content with the addition of 0.8 wt.% Ca, so the peak intensity is weak. With the further increment of Ca content, the characteristic peak intensity is obviously stronger at 1 wt.% Ca. Therefore, it can be determined that the precipitated new phase is the CaMgSi phase. There have been many studies on the mechanism of modification of the Mg_2_Si phase with Ca in magnesium alloys. In general, there are two viewpoints for the mechanism: the poisoning effect [24] and the effect of heterogeneous nucleation [25]. Ca atoms could absorb in the Mg_2_Si crystal plane during solidification. This would cause constitutional undercooling at the solidification interface, and reduce the surface energy of the Mg_2_Si crystals. As a result, the growth habit of the Mg_2_Si phase has been changed. This is the poisoning effect. In this study, the enrichment of Ca in the core of the primary Mg_2_Si phase was not found. Therefore, the mechanism of modification is mainly the poisoning effect.

**Figure 9:**
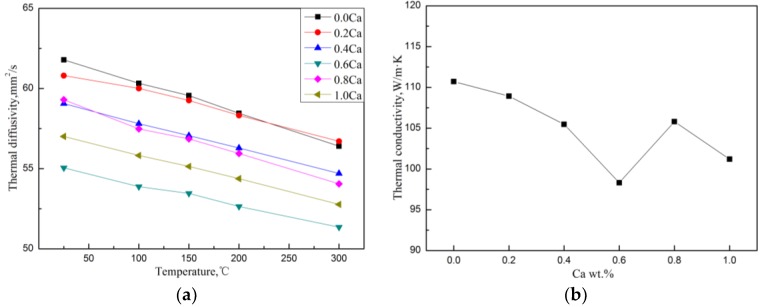
Thermal diffusion (**a**) and room temperature thermal conductivity (**b**) of Mg–4Si–yCa alloy.

The thermal diffusivity in the temperature range of 25–300 °C and the room temperature TC of the Mg–4Si–yCa alloy are shown in Figure 9. It was found that when the Ca content is less than 0.8 wt.%, the TC of the alloy decreases gradually with the increase of Ca content. Table 4 shows the volume fraction of the second phase in the Mg–4Si–yCa alloys. It is shown that the volume fraction of the Mg_2_Si phase in the alloys decreases gradually with the increase Ca content (<0.8 wt.% Ca). This phenomenon indicates that the addition of Ca element inhibits the growth of the Mg_2_Si phase. So, the amount of precipitated Mg_2_Si phase decreases, and the Si content in the Mg matrix increases. The reduction of the phase interface could enhance the TC of the alloy, while the increase of the solid solution atoms in the matrix could cause a decrement in the TC. According to the results shown above (Figure 9), it seems like the change of the amount of solid solution atoms plays a major role on the TC of the alloy. When the addition of Ca content reaches 0.8 wt.%, the CaMgSi phase forms in the alloy. The precipitated new phase consumes Ca and Si elements in the alloy, which leads to the decrement of the solid solution atoms in the matrix and the increment of the number of the second phase. Therefore, the TC of the alloy has a significant improvement. The further addition of the Ca element increases the number and the size of precipitated CaMgSi phases. This introduces some new phase interfaces, and excess Ca will also solidify into the matrix, causing the TC of the alloy to decrease again.

## 4. Conclusions

The thermal conductivities of Mg–xSi (x = 1 wt.%, 1.38 wt.%, 2 wt.%, 3 wt.%, and 4 wt.%) alloys and Mg–4Si–yCa (y = 0.2 wt.%, 0.4 wt.%, 0.6 wt.%, 0.8 wt.%, and 1.0 wt.%) alloys in the temperature range of 25–300 °C were studied, and the conclusions were as follows:

(1) The TC of Mg–Si binary alloys with different Si content is systematically studied for the first time. When the Si content increases, the TC of Mg–Si binary alloys decreases gradually, but there is a slight increase at the eutectic composition, which originates from the formation of a small amount of primary Mg_2_Si phase that reduces the amount of eutectic Mg_2_Si phase.
(2) The TC of the as-cast Mg-1%Si alloy is 124.7 W/(m·K). The TC of the Mg-1%Si binary alloy is lower than that of the Mg-1%Zn binary alloy, but it is better than the Mg-0.9%Al binary alloy. It is mainly because of the lower solid solubility of Si in Mg, and the formation of more phase interfaces than in the Zn case.
(3) The addition of Ca has an obvious modification effect on Mg_2_Si phase. With the addition of Ca, the morphology of primary Mg_2_Si phase changes from coarse dendritic to small polygonal particles, and the distribution of the eutectic Mg_2_Si phase becomes discontinuous.
(4) With the increment of Ca content, the TC of Mg–4Si–yCa alloys decreases gradually. When the Ca content is 0.8 wt.%, the TC of the alloy is obviously improved. This is due to the formation of the CaMgSi phase, which consumes the Ca and Si elements in the α–Mg matrix and releases the lattice distortion of the matrix. When further increasing the Ca content, the excess Ca element solves into the α–Mg matrix, which causes the TC of the alloy to decrease again.

## Figures and Tables

**Table 1:** Room temperature density and calculation of the specific heat capacity of Mg–Si binary alloys.

Alloys	Density (g/cm^3^)	C_p_ (J/g·K)
Mg-1Si	1.757	1.023
Mg-1.38Si	1.758	1.022
Mg-2Si	1.760	1.020
Mg-3Si	1.761	1.017
Mg-4Si	1.767	1.014

**Table 2:** Room temperature density and calculation of specific heat capacity of Mg–4Si–yCa alloys.

Alloys	Density (g/cm^3^)	C_p_ (J/g·K)
Mg-4Si-0.2Ca	1.765	1.015
Mg-4Si-0.4Ca	1.761	1.014
Mg-4Si-0.6Ca	1.763	1.013
Mg-4Si-0.8Ca	1.763	1.012
Mg-4Si-1.0Ca	1.756	1.011

**Table 3:** The second phase volume fraction of Mg–Si binary alloy.

Alloy	Mg–1Si	Mg–1.38Si	Mg–2Si	Mg–3Si	Mg–4Si
**Volume Fraction (%)**	30.72	29.59	37.97	40.70	46.50

**Table 4:** Volume fraction of second phase of Mg–4Si–yCa alloy.

**Ca Content (wt.%)**	0	0.2	0.4	0.6	0.8	1.0
**Volume Fraction (%)**	46.49	41.41	31.61	26.46	35.88	32.65
